# A Case of Extensive Airway Necrosis Following Esophagectomy Successfully Treated with Airway Stenting

**DOI:** 10.3390/clinpract15120223

**Published:** 2025-11-27

**Authors:** Tatsuki Tsuruga, Hajime Fujimoto, Esteban C. Gabazza, Masaki Ohi, Masahide Oki, Tetsu Kobayashi

**Affiliations:** 1 Department of Pulmonary and Critical Care Medicine, Faculty and Graduate School of Medicine, Mie University, 2-174 Edobashi, Tsu 514-8507, Mie, Japan; y.o.spriggan@gmail.com (T.T.);; 2 Department of Immunology, Mie University Graduate School of Medicine, Tsu 514-8507, Mie, Japan; 3 Department of Gastrointestinal and Pediatric Surgery, Mie University Graduate School of Medicine, Tsu 514-8507, Mie, Japan; 4 Department of Respiratory Medicine, National Hospital Organization (NHO) Nagoya Medical Center, Nagoya 460-0001, Aichi, Japan

**Keywords:** esophageal cancer, esophagectomy, bronchial artery ligation, airway necrosis, bronchoscopy, airway stent, postoperative complication

## Abstract

Background: Airway stenting is an alternative therapy for patients with complicated esophagectomy. Case presentation: A 60-year-old man with clinical stage IIIA esophageal cancer underwent neoadjuvant chemotherapy followed by robot-assisted subtotal esophagectomy with cervical esophagogastrostomy and jejunostomy. During surgery, both bronchial arteries were ligated to facilitate esophageal mobilization. Bronchoscopy on the first postoperative day showed no abnormalities; however, by the second postoperative day, the patient developed pneumonia and septic shock, requiring mechanical ventilation. On the fifth postoperative day, bronchoscopy revealed extensive epithelial injury extending from the trachea to both main bronchi, indicating ischemic airway damage. He was diagnosed with airway necrosis and referred to our respiratory department. Serial bronchoscopic examinations and suctioning of the sloughed epithelium were performed, and a tracheostomy enabled weaning from mechanical ventilation. By the twenty-fourth postoperative day, bronchoscopy revealed the accumulation of large, hardened secretions within the trachea, carina, and both main bronchi, resulting in airway narrowing and a high risk of asphyxiation. A silicone Y-shaped airway stent was inserted to maintain patency. Following stent placement, airway secretions progressively decreased, and the patient was discharged on the sixty-third postoperative day. The stent was removed six months later, with no recurrence of airway or respiratory complications. Conclusion: This case illustrates a rare but severe complication of extensive airway necrosis, likely caused by intraoperative bronchial artery ligation and dissection of the tracheal membranous portion. Although preservation of the bronchial arteries and meticulous surgical technique are essential preventive strategies, such complications may be unavoidable. In cases of extensive airway necrosis, airway stenting can serve as an effective therapeutic option to prevent obstruction and support recovery.

## 1. Introduction

Airway necrosis following esophagectomy for esophageal cancer is a serious complication [1,2,3]. Risk factors include intraoperative ligation of the bronchial arteries and preoperative radiation therapy [3,4]. While management of airway necrosis varies with severity, airway stenting is used infrequently because the radial force of the stent may further compromise mucosa with already reduced blood flow and potentially exacerbate ischemia. Alternative approaches include optimized conservative management, tracheostomy to facilitate secretion clearance, and, in selected cases, placement of a Montgomery T-tube, which exerts less radial pressure than conventional stents. [5]. Airway necrosis is thought to result from disruption of the bronchial circulation, which provides the primary vascular supply to the tracheobronchial tree. During esophagectomy, primarily when performed via a transthoracic or robotic-assisted approach, the bronchial arteries are frequently ligated to allow sufficient dissection around the esophagus [6,7,8]. The resulting ischemia may be exacerbated by systemic hypoperfusion, as in cases complicated by septic shock or poor perioperative circulatory management [9,10]. In addition, prior chemoradiotherapy can impair microvascular integrity and tissue repair, further predisposing patients to ischemic injury [11,12]. Although conservative management is generally preferred, severe cases with extensive necrosis and impending airway compromise may require timely intervention, including stenting or surgical repair [9]. However, the role of airway stenting remains controversial, as stents may exacerbate ischemia and can introduce complications, including granulation tissue formation, stent migration, and mucus plugging. Consequently, the decision to place a stent should be made on a case-by-case basis after carefully weighing risks and benefits [13].

Herewith, we report a rare case of extensive airway necrosis developing after esophagectomy for esophageal cancer, involving the trachea and both main bronchi. As the patient subsequently developed a high risk of airway obstruction due to the accumulation of extensive crusts, a silicone stent was inserted. Following stent placement, tracheal secretions gradually decreased, and the stent was safely removed after confirming improvement of the airway necrosis. This case highlights that silicone stenting may be an effective therapeutic option for extensive airway necrosis, particularly when there is concern for airway obstruction.

## 2. Case Report

A male patient was admitted to our institution with a diagnosis of clinical stage IIIA esophageal cancer. His medical history was notable for chronic obstructive pulmonary disease, with a forced expiratory volume in one second (FEV1) of 2.84 L and a percentage of predicted FEV1 of 88.5%. The FEV1.0% was 68.4%. He was undergoing inhalation therapy with tiotropium bromide and olodaterol hydrochloride. The patient had a substantial smoking history of 84 pack-years. He also reported a daily alcohol intake equivalent to approximately 400 mL of Japanese sake.

**Figure 1:**
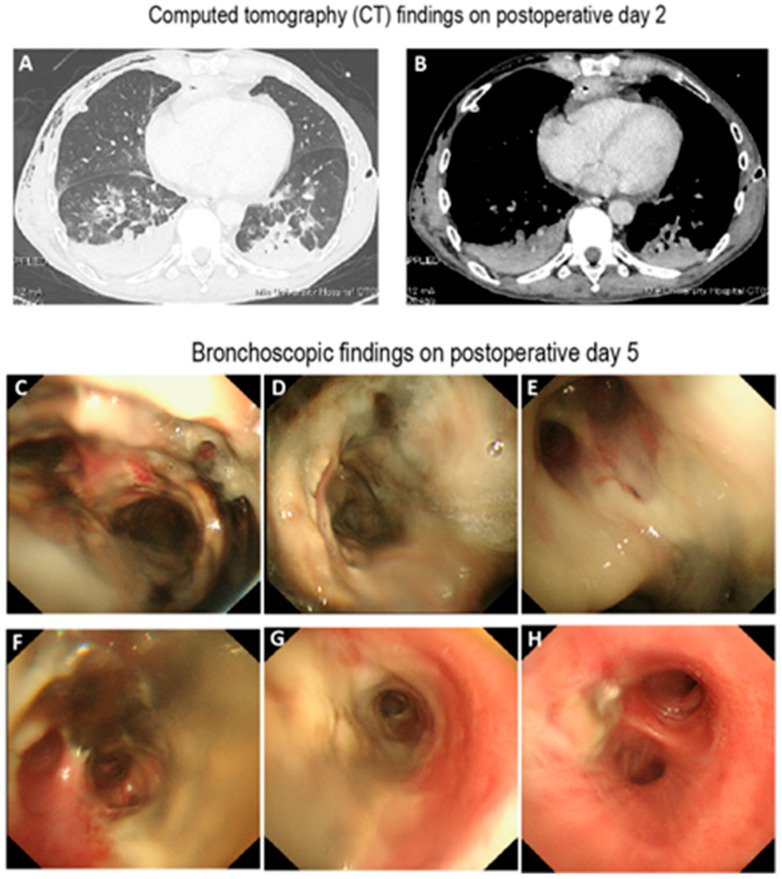
Postoperative computed tomography and bronchoscopic findings. (**A,B**) Computed tomography image obtained on postoperative day 2, demonstrating pneumonia, atelectasis, right-sided pneumothorax, and subcutaneous emphysema. These findings necessitated the placement of a right-sided chest tube. (**C**–**H**) Bronchoscopic images on postoperative day 7 showing widespread epithelial whitening involving the trachea (**C**), right main bronchus (**D**), orifice of the right upper lobe bronchus (**E**), bronchus intermedius (**F**), right lower lobe bronchus, and the posterior wall of the left main bronchus (**G**). Focal black discoloration was also observed. In contrast, the epithelium distal to the left secondary carina appeared unremarkable.

His esophageal cancer was treated with neoadjuvant chemotherapy with cisplatin and 5-fluorouracil, followed by robot-assisted subtotal esophagectomy, cervical esophagogastrostomy using a retrosternal route, and jejunostomy. During the procedure, both bronchial arteries were ligated as part of the esophageal mobilization. Bronchoscopy on postoperative day 1 revealed no significant abnormalities in the tracheal or bronchial epithelium. However, on postoperative day 2, the patient developed pneumonia and septic shock, requiring intensive systemic management including invasive mechanical ventilation as demonstrated by the postoperative CT findings (Figure 1A,B) and the results of the laboratory blood test (Table 1). Mechanical ventilation was required until postoperative day 14. On postoperative day 3, bronchoscopy showed mild erythema and subtle dark discoloration of the right main bronchial epithelium. By postoperative day 5, the bronchoscopic study showed widespread epithelial damage, suggestive of impaired blood flow from the trachea to both main bronchi, leading to a diagnosis of airway necrosis (Figure 1C–H). The patient was then referred to our Pulmonary Department.

**Figure 2:**
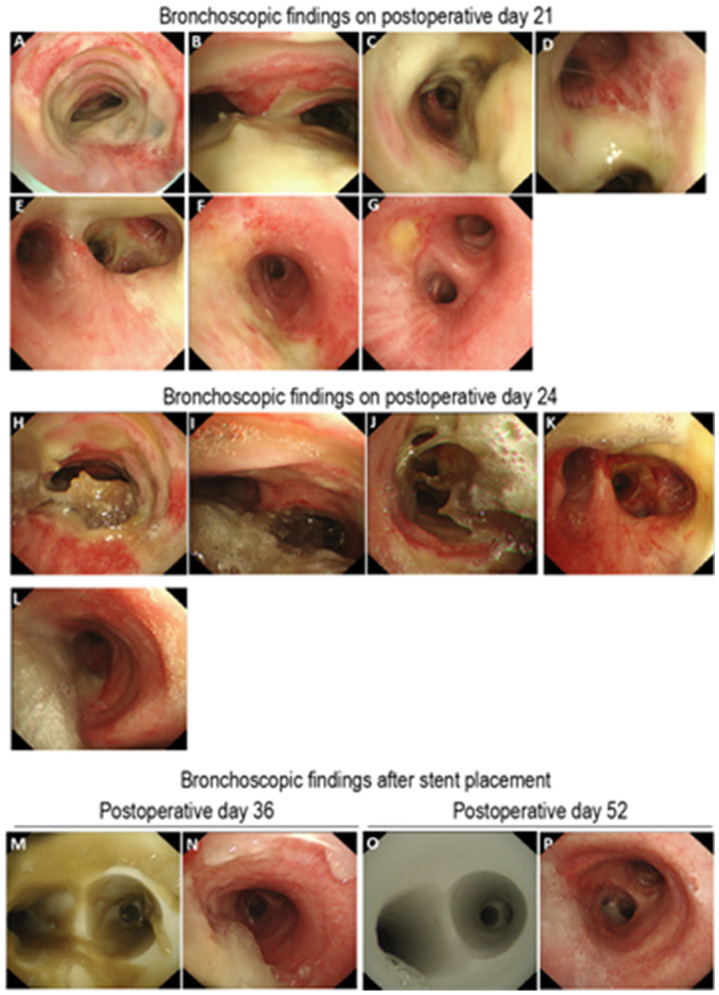
Serial bronchoscopic findings following esophagectomy. (**A**–**G**) Bronchoscopy performed on postoperative day 21 revealed findings in the trachea (**A**), tracheal carina (**B**), right main bronchus (**C**), orifice of the right upper lobe bronchus (**D**), right bronchus intermedius (**E**), left main bronchus (**F**), and left secondary carina (**G**). A notable improvement in mucosal appearance was observed. The previously noted black discoloration and epithelial whitening had diminished, and bronchial secretions were reduced, indicating a trend toward healthier epithelial coloration. (**H**–**L**) Bronchoscopy performed on postoperative day 24 demonstrated extensive, hardened secretions adhering to the walls of the trachea (**H**), tracheal carina (**I**), right main bronchus (**J**), right bronchus intermedius (**K**), and left main bronchus (**L**). (**M**–**P**) Serial findings following silicone stent placement. On postoperative day 36, bronchoscopy revealed abundant yellowish secretions within the stent lumen in the trachea (**M**) and left main bronchus (**N**). By postoperative day 52, the secretions had markedly decreased, as seen in the tracheal carina (**O**) and left main bronchus (**P**), reflecting improved airway patency.

The patient underwent upper gastrointestinal endoscopy and CT, but these studies did not reveal findings suggestive of anastomotic leakage or mediastinal abscess, making secondary airway necrosis unlikely. Regular bronchoscopic assessments, including suctioning of airway secretions, were performed in conjunction with systemic supportive care. The patient’s overall condition gradually improved. Vasopressors were discontinued on postoperative day 8, and antibiotics were discontinued on postoperative day 20. Bronchoscopic image on postoperative day 21 showed improvement in airway epithelial integrity (Figure 2A–G).

**Figure 3:**
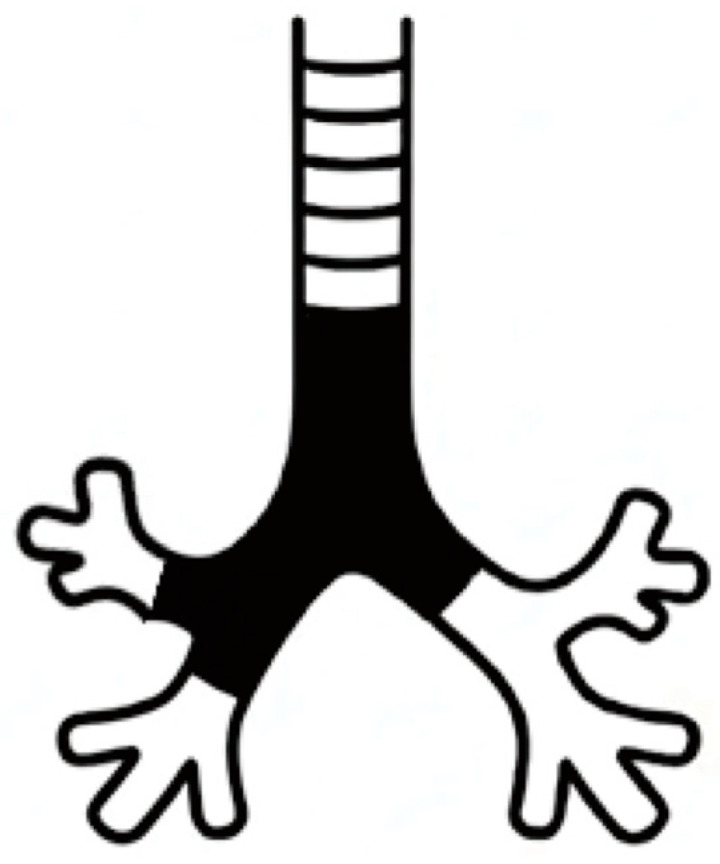
Schematic illustration of the airway stent placement. A silicone Y-stent was positioned across the distal trachea and carina, extending into both main bronchi to maintain airway patency and prevent obstruction from sloughing necrotic tissue.

However, bronchoscopy on postoperative day 24 revealed extensive and dense crusting across the airway epithelium (Figure 2H–L). Daily bronchoscopic debridement of the crusts was initiated; however, owing to the risk of airway obstruction and asphyxiation, a silicone Y-stent was placed on postoperative day 31 at another hospital specializing in bronchoscopic interventions. Stent placement was performed under general anesthesia using a rigid bronchoscope. The silicone Y-stent extended from the trachea to the carina, both main bronchi, and the right upper lobe bronchus (Figure 3). Following stent placement, nebulized therapy with acetylcysteine, bromhexine, and procaterol was administered, resulting in a gradual decrease in airway secretions (Figure 2M–P). The patient’s condition stabilized, and he was discharged home on postoperative day 63.

Approximately seven months after surgery, six months after the airway stent had been placed, the patient exhibited impaired clearance of bronchial secretions attributable to the indwelling stent. The device was therefore electively removed, and respiratory function remained stable thereafter. Imaging performed at the same postoperative time point, however, disclosed locoregional recurrence of esophageal carcinoma. Salvage therapy with immune-checkpoint inhibitors failed to confer clinical benefit, and the patient ultimately succumbed to disease progression 12 months after the initial operation.

## 3. Discussion

Airway necrosis following esophagectomy is a rare but serious complication, reported to occur in 0.2% to 7.0% of cases [3]. Based on its underlying mechanisms, airway necrosis is broadly classified into primary and secondary types. Primary airway necrosis is primarily caused by ischemia resulting from direct surgical disruption of the tracheobronchial blood supply. In contrast, secondary airway necrosis results from infectious processes, such as anastomotic leakage or cervical/mediastinal abscesses, which extend to the airways and induce necrosis through inflammation. In the present case, postoperative CT and upper gastrointestinal endoscopy revealed no evidence of anastomotic leakage, mediastinitis, or abscess formation, leading to a diagnosis of primary airway necrosis.

The severity of airway necrosis is commonly assessed using the classification proposed by Sakai et al. [3]. This system categorizes cases into three grades: Grade 1 involves only mucosal necrosis; Grade 2 includes necrosis extending into the muscular layer without fistula or perforation; and Grade 3 refers to full-thickness necrosis accompanied by fistula or perforation. In their report, Grade 1 was the most frequent (62.5%), whereas Grades 2 and 3 were significantly associated with lower preservation rates of the bronchial arteries (BAs). In our case, although extensive necrosis was observed from the trachea to the bilateral main bronchi, intermediate bronchus, and right lower lobe bronchus, no evidence of fistula or perforation was present. Thus, this case was considered Grade 2.

Established risk factors for airway necrosis include preoperative radiotherapy and disruption of the bronchial arteries [7]. Prior studies have shown that tracheal blood flow may be reduced by up to 70% following chemoradiotherapy [14] and that bronchial artery clamping significantly reduces tracheal epithelial perfusion [15]. Other suggested risk factors include posterior mediastinal reconstruction and comorbid diabetes mellitus [7,16].

Tracheobronchial perfusion is primarily sustained through collateral circulation originating from the bronchial arteries, inferior thyroid arteries, and subclavian arteries. Preservation of the bronchial arteries during esophagectomy is crucial for preventing tracheobronchial ischemia and preserving postoperative respiratory function. However, during esophageal mobilization, especially around the right side, preservation of the right bronchial artery is often not feasible [17,18]. Furthermore, procedures such as tracheostomy, thyroidectomy, and lymphadenectomy around the trachea can further compromise tracheal blood flow.

In the present case, bilateral bronchial artery transection, combined with postoperative septic shock-induced circulatory failure, was considered the primary factor contributing to the extensive airway necrosis observed. Although the patient had no history of preoperative radiotherapy or diabetes, the combination of bilateral bronchial artery disruption and systemic hypoperfusion due to septic shock likely exacerbated the ischemic necrosis. Tracheal intubation and excessive cuff pressure are also known risk factors for airway necrosis [19]. While cuff pressures between 20 and 30 cm H_2_O are generally recommended, both underinflation and overinflation are common in clinical practice, with studies reporting cuff underinflation in 54% and overinflation in 73% of patients [20]. At our institution, we routinely use cuff pressure manometers to ensure appropriate management.

According to Sakai et al., the median time to diagnosis of airway necrosis is 6 days for Grade 1, 9 days for Grade 2, and 117 days for Grade 3, with the majority of cases (except Grade 3) diagnosed within 30 days postoperatively [3]. In our case, airway necrosis was also diagnosed in the early postoperative period. This highlights the critical importance of closely monitoring respiratory status and airway secretions, as well as conducting early bronchoscopic examinations for timely diagnosis.

Regarding prognosis, most Grade 1 cases respond well to conservative management. However, approximately 70% of Grade 2 cases require surgical intervention, and many Grade 3 cases fail to achieve resolution of necrosis [3]. With appropriate management, the survival rate of Grade 1 patients may be comparable to that of patients without airway necrosis.

A notable feature of the present case was the unusually extensive necrosis involving the trachea, both main bronchi, the intermediate bronchus, and the right lower lobe bronchus. To our knowledge, while localized or segmental airway necrosis has been previously reported [16], there are no prior reports of airway necrosis extending to such an extensive area as in the present case. Another important aspect was the prompt improvement of inflammatory markers with multidisciplinary management, including antimicrobial therapy, with bronchoscopic toileting likely contributing to infection control. No elevation in inflammatory parameters was observed before stent placement, nor was any increase detected after its insertion. Importantly, complications typically associated with impaired airway clearance following stenting, such as increased inflammatory response, difficulty expectorating, or secondary airway infection, did not occur in this case.

In general, stent placement may exacerbate ischemic injury by exerting pressure on compromised tissues and has been associated with worsening necrosis [16]. Additionally, high-positioned stents in patients with cervical esophageal reconstruction may cause significant discomfort [16]. However, in our case, the risk of airway obstruction was extremely high due to the formation of large necrotic crusts, and stent placement was necessary to improve respiratory status and secure airway clearance. Several factors likely contributed to the success of stent therapy in this case: (1) the stent was placed after a delay from onset, during a period when epithelial regeneration and partial revascularization may have occurred; (2) the pathology was Grade 2 necrosis without fistula or perforation despite its extensive distribution; and (3) the absence of preoperative radiotherapy and diabetes. These factors may have limited further necrotic progression after stent placement and contributed to the favorable outcome. Nevertheless, the optimal indications and timing for stent placement in airway necrosis remain unclear and warrant further investigation and accumulation of clinical cases.

## 4. Conclusions

In conclusion, this is a rare case of extensive tracheobronchial necrosis following esophagectomy that was successfully managed with silicone stent placement. Bilateral bronchial artery transection and circulatory failure due to septic shock were likely central to the development of widespread airway necrosis. Prevention of airway necrosis requires a multidisciplinary and comprehensive approach, including maximal preservation of the bronchial arteries, rigorous perioperative circulatory management, and careful consideration of the indications and radiation fields in preoperative radiotherapy. Based on our experience, in cases of extensive airway necrosis, particularly when there is a high risk of airway obstruction, silicone stent placement may serve as an effective therapeutic option, provided its use is carefully indicated. Future efforts should focus on the early diagnosis of airway necrosis, identifying high-risk patients, and developing optimal treatment strategies, including the role of stenting.

## Figures and Tables

**Table 1:** Laboratory findings.

Parameters	Value	Normal Range
WBC	8.65 × 10^3^	4.0–10.0 × 10^3^/μL
Neu	92	40–75%
Lym	2.99	20–45%
Mono	2.99	2–8%
Eos	0	1–4%
Bas	0	0–1%
RBC	3.39 × 10^6^	4.5–5.9 × 10^6^/μL
Hb	12.2	13.5–17.5 g/dL
Ht	35.7	41–53%
Plt	2.50 × 10^5^	150–400 × 10^3^/μL
Albumin	2	3.5–5.0 g/dL
T-bil	0.7	0.2–1.2 mg/dL
BUN	16.8	7–20 mg/dL
Creatinine	0.78	0.7–1.3 mg/dL
Sodium	124	135–145 mEq/L
Potassium	4.4	3.5–5.0 mEq/L
Chloride	96	98–107 mEq/L
AST	22	8–38 IU/L
ALT	12	4–44 IU/L
LDH	199	120–250 IU/L
Glucose	129	70–110 mg/dL (fasting)
HbA1c	5	4.0–5.6%
CRP	22.2	<0.3 mg/dL
BNP	52.6	<100 pg/mL
PT	14.8	11–15 s
PT%	60	70–130%
APTT	39.7	25–35 s
D-dimer	8	<1.0 μg/mL
Fib	691	200–400 mg/dL

WBC, white blood cells; Neu, neutrophil; Lym, lymphocyte; Mono, monocyte; Eos, eosinophil; Bas, basophil; RBC, red blood cells; Hb, hemoglobin; Ht, hematocrit; Plt, platelets; T-bil, total bilirubin; BUN, blood urea nitrogen; AST, aspartate aminotransferase; ALT, alanine aminotransferase; LDH, lactate dehydrogenase; HbA1c, hemoglobin A1c; CRP, C-reactive protein; BNP, brain natriuretic peptide; PT, prothrombin time; APTT, activated partial thromboplastin time; Fib, fibrinogen.

## Data Availability

All data supporting the findings of this study are included in this report. Additional information can be obtained from the first author upon reasonable request.
